# Developments and Challenges in Durable Ventricular Assist Device Technology: A Comprehensive Review with a Focus on Advancements in China

**DOI:** 10.3390/jcdd11010029

**Published:** 2024-01-18

**Authors:** Jingrong Tu, Li Xu, Fei Li, Nianguo Dong

**Affiliations:** 1 Department of Cardiovascular Surgery, Union Hospital, Tongji Medical College, Huazhong University of Science and Technology, 1277 Jiefang Ave, Wuhan 430022, China; tjrforwork@126.com (J.T.); 2023xh0103@hust.edu.cn (L.X.); 2 Fuwai Yunnan Cardiovascular Hospital, Kunming Medical University, 528 Shahebei Rd, Kunming 650500, China

**Keywords:** heart failure, mechanical circulatory support, durable ventricular assist devices, complications

## Abstract

Heart transplantation is currently the most effective treatment for end-stage heart failure; however, the shortage in donor hearts constrains the undertaking of transplantation. Mechanical circulatory support (MCS) technology has made rapid progress in recent years, providing diverse therapeutic options and alleviating the dilemma of donor heart shortage. The ventricular assist device (VAD), as an important category of MCS, demonstrates promising applications in bridging heart transplantation, destination therapy, and bridge-to-decision. VADs can be categorized as durable VADs (dVADs) and temporary VADs (tVADs), according to the duration of assistance. With the technological advancement and clinical application experience accumulated, VADs have been developed in biocompatible, lightweight, bionic, and intelligent ways. In this review, we summarize the development history of VADs, focusing on the mechanism and application status of dVADs in detail, and further discuss the research progress and use of VADs in China.

## 1. Introduction

Cardiovascular diseases (CVDs) are ranked as the first killer in the United States [1]. Heart failure (HF) is the end-stage symptom of various kinds of chronic CVDs, resulting from the accumulating cardiac impairments of the heart’s structure [2,3]. The prevalence of HF is continually increasing and reaches approximately 4.5 million cases in China [4]. Heart transplant is regarded as the gold standard therapy, but the lack of donor hearts severely limits the application [5]. Recent advancements in mechanical circulatory support (MCS) technology have significantly broadened the range of therapeutic choices available. This progress shows this technology is an instrumental tool in managing severe heart conditions, offering alternative solutions for patients with advanced HF, bridging the gap between medical therapy and heart transplantation [6]. The application of ventricular assist devices (VADs) has revolutionized the management of heart failure, particularly for patients ineligible for heart transplantation or awaiting a donor heart.

According to the duration of application, VADs could be divided into temporary VADs (tVADs) and durable VADs (dVADs) [7]. tVADs are commonly used for acute HF patients to maintain the stability of circulation in a short time, no more than several weeks and months in most cases. Several systems, including Hemopump™ (Johnson&Johnson), ABIOMED, and Impella, in recent practice, have received great attention and have considerable efficacy [8,9]. dVADs can be implanted into the thoracic cavity of patients and support cardiac function in the long term, with its duration ranging from several months to several years.

The introduction of next-generation VADs has enabled longer term support, with a reduced morbidity associated with heart failure and significantly improved patient outcomes [10,11,12]. By examining recent studies and clinical reports, this review seeks to summarize the recent advancements in dVAD technology and their current utilization status around world and especially in China, offering insights into its impact.

## 2. Historical Development of Durable Ventricular Assist Devices

The initial strategy towards end-stage HF centered around complete heart replacement, focusing on total artificial hearts (TAHs). However, the launch of the United States’ Artificial Heart Program in 1962 illustrated a dramatic shift in therapeutic approach [13]. The focus was pivoted on augmenting the heart’s existing capabilities, leading to the development of single-chamber pump assist devices and eventually VADs [14].

The inception of VAD technology was marked by the implantation of the first gas-energized, synchronized, hemispherical pump by DeBakey and Liotta on 19 July 1963 in Houston [15]. However, the significant milestone in VAD history was the successful execution of the first VAD-Bridge to Transplant (VAD-BTT) surgery practiced with the Novacor device in 1984 [14].

### 2.1. First-Generation dVADs

The first generation of dVADs partially inherited the idea of artificial heart replacement therapies, whereby a pulsatile blood flow was generated by a pneumatic or electric drive to assist ventricular function, being represented by the Pierce–Donachy VAD (conducted by Thoratec Laboratories Corporation in California from 1985), Novacor LVAS (conducted by Baxter Healthcare Division in 1988), and HeartMate VEX (also known as HeartMate I, approved by the FDA in 1994) [16,17]. The Thoratec PVAD followed a similar way of design and was put into practice in 1995 [18]. Despite being innovative, its technological immaturity brought constraints in application. More than the expected performance, the first-generation dVADs had significant issues, such as large size, uncontrollable noise, and malfunction due to the tearing of the power unit envelope or degradation of valves [19]. These drawbacks led to various complications, severely reducing the quality of life of patients after implantation and notably increasing the risk of post-discharge from the hospital, making the use of first-generation dVADs highly hospital-dependent.

### 2.2. Second-Generation dVADs

The second generation of dVADs used axial flow centrifugation to generate a continuous flow (CF) for circulation, being represented by the HeartMate II, JARVIK 2000, and Debakey by MicroMed Cardiovascular Inc. from Houston, TX, USA [20,21]. The reduced size and increased biocompatibility effectively improved patient prognosis, and the application of impeller mechanics and magnetic fixation combining technology in this generation significantly increased the device durability [22]. Moreover, the significantly reduced noise level improved the quality of life of patients after discharge. The HeartMateII, the most widely used device of second-generation dVADs, was approved by the FDA for use in BTT in 2008 and later for use in DT in 2010. Data from a clinical study comparing first- and second-generation dVADs showed that the use of the continuous flow (CF) technology of second-generation dVADs had a significant advantage over first-generation dVADs using a pulsatile flow technology in terms of improved postoperative survival and reduced complication rates [23]. As of 2017, the share of CF-dVAD implanted devices, including third-generation dVADs, has exceeded 95% [23].

However, the second generation also suffered from some design defects. The continuous flow pump of the second-generation dVAD used a rigid bladed rotor shaft, although it was able to meet the ejection demands by rotating at high speeds; the high-speed rotor shaft was prone to heat production when in contact with the bloodstream, which caused the destruction of blood components and the formation of blood clots, increasing the risk of thromboembolism. With the widespread use of the HeartMate III, the HeartMate II was retired from the market in 2019.

### 2.3. Third-Generation dVADs

In the third generation of dVADs, represented by the EvaHeart and HeartMate III, the use of magnetic or hydrodynamic levitation fixation technology was merged with higher pumping efficiencies, allowing the third-generation dVADs to meet the pumping demands at lower rotational speeds, significantly reducing the destruction of blood components.

The EVAHEART design was initiated in 2002 and was later approved for clinical use for BTT by the Ministry of Health, Labor, and Welfare of Japan for manufacturing and sale in 2010. It was the first to utilize an open-impeller fluid dynamic suspension system and retrieved excellent clinical outcomes domestically. The significant lower occurrence of complications, including right heart failure and gastrointestinal bleeding, enabled its acquisition by the U.S. Medical Device Investigational Device Exemption (IDE) and European Conformity (CE) regulations.

HeartMate III is the world’s first artificial heart with full magnetic levitation technology. The first clinical implantation was launched in 2014, approved for BTT in 2017, and approved for DT in 2018. It is at present the most widely used VAD in the world, with 80% of the market in the U.S. [24]. The unique FullMagLev magnetic levitation technology of HeartMate III effectively reduces wear and heat generation, and this “no wear-out” technology makes it last for more than a decade. The continuous rotation of the levitated rotor avoids the stagnation of blood flow within the ventricle, and the non-contact design of the rotor and the mechanical ventricle significantly reduces the disruption of blood components, which in turn reduces the risk of hemorrhagic or thrombotic complications [25]. In 2022, a multicenter study (MOMENTUM 3) on the use of magnetic levitation technology for patients receiving HeartMate mechanical circulatory support therapy compared the five-year prognosis of HeartMate II and III on the line. The results revealed that the latter had significant advantages in BTT, DT, and BTD and a lower incidence of serious adverse events, such as strokes, hemorrhage, and thrombosis [26].

## 3. Advancement and Future Perspectives of Durable Ventricular Assist Device Engineering

The development of dVADs, particularly third-generation dVADs, represents a remarkable convergence of advanced engineering and clinical innovation. This critical technology for advanced HF patients has gained significant development in reducing the risk of hemolysis and thrombosis with the advent of magnetic or hydrodynamic-levitated rotors.

One important aspect of the advancement in dVADs is the focus on blood compatibility. The non-Newtonian nature of the blood requires careful consideration in dVADs design to minimize the potential for blood cell damage and subsequent complications, including thrombosis, bleeding, and acquired vascular pseudohemophilia [27]. For example, the HeartWare Ventricular Assist Device (HVAD) utilizes a combination of magnetic and hydrodynamic forces to levitate its rotor, and HeartMate III utilizes a fully magnetically levitated rotor. These characteristics significantly reduce mechanical wear and improve the life of the device. Another important aspect of these devices is their ability to regulate flow. This feature is particularly important for controlling the formation of microthrombi. In practice, HeartMate III uses programmable algorithms to regulate the rotor speed to introduce a degree of pulsation that reduces the risk of cardiovascular and hematologic complications common in a non-pulsatile flow. The CorWave LVAD and TORVAD developed novel approaches to momentum transfer to minimize blood trauma, probably offer a promising solution to microthrombi [28,29]. Nevertheless, long-term clinical investigation is required to confirm their overall safety and stability.

Despite advances, challenges remain in the deploying of the devices. The risk of infection at the driveline site and the need for permanent skin breakdown are yet to be addressed satisfactorily and pose great concerns to the dVADs’ recipients’ prognosis. Developing a fully implantable system without a driveline can be a solution. The HVAD has provided progress in limiting the size of the device and has been successfully used in minimally invasive thoracotomy [30]. However, it was recalled in 2021 due to the significantly higher percentage of long-term nervous adverse events after implantation [31].

Future developments in dVAD technology may focus on further reducing thrombosis through surface modification and exploring less invasive impeller geometries. In addition, the increase in pump flow through exercise to accommodate the body’s varying metabolic demands may be a potential direction to elevate life quality after implantation. Artificial intelligence integration to optimize pump parameters based on real-time data may be another area ripe for exploration.

## 4. Current Status of Durable Ventricular Assist Device Application

The utilization and strategy of dVADs have significantly evolved and expanded in recent years. A total of 3198 patients with advanced HF received continuous flow (CF) durable left ventricular assist devices in the U.S. in 2019, a peak in the history of the Society of Thoracic Surgeons Intermacs Registry [32].

Compared to the earlier half of the decade, patients implanted with dVADs from 2015 to 2019 presented with more comorbidities and social challenges, which means that there are larger numbers in the population that are less likely to be direct candidates for transplantation [33]. Notably, more than half of the recipients during this period were in some degree of cardiogenic shock at the time of implantation [24], and there was a significant rise in preoperative temporary MCS usage. These trends are consistent globally, underscoring a universal shift in the landscape of advanced HF management. Historically, DT dVADs were likely to be pursued in patients ineligible for heart transplantation due to factors like age, comorbidities, or psychosocial issues. Patients eligible for transplantation were usually considered for dVADs in BTT cases. However, a decisive shift occurred in recent years, with over 70% of dVAD implantations in 2019 being for DT and less than 10% for BTT [33]. The proportion of patients receiving VADs for DT in Europe and the United States was 19.1% in 2000 and rose to 40% in 2012 [34]. As of 2017, in Europe and the United States, the number of patients receiving VAD support continued to increase, and the use for DT reached 50%, compared to 26% for BTT [35]. This trend likely stems from the U.S. FDA device labeling for DT support, improved outcomes after dVAD implantation, and revisions in the 2018 United Network for Organ Sharing (UNOS) donor heart allocation system [36].

Despite this shift, there is a notable disparity in survival probabilities between patients implanted with DT dVADs and BTT dVADs, with the former showing slightly lower survival rates at two years [37]. This disparity is likely due to the greater comorbidity burden among DT dVAD recipients and the inclusion of patients with older devices in the registry. However, advancements in technology may narrow the gap. The MOMENTUM 3 study with HeartMate III demonstrated comparable outcomes at two years, regardless of the implantation intent [26]. These findings suggest that the distinction between DT and BTT may be blurring in the current landscape, as some patients initially receiving DT dVADs may become eligible for transplantation and vice versa.

In summary, the current status of dVAD usage is marked by a record-high volume of implantations, a shift in patient demographics towards those with more comorbidities and social challenges, a strategic shift from BTT to DT, and a consolidation in the types of devices used. This evolution reflects both technological advancements and changes in clinical practice, highlighting the dynamic nature of advanced HF management and the critical role of dVADs in this spectrum.

## 5. Challenges and Management Strategies of Adverse Events in Durable Ventricular Assist Devices

The remarkable advancements in dVADs have transformed the landscape of heart failure management, yet they come with a spectrum of adverse events that pose significant challenges for both patients and healthcare providers. Understanding and managing these complications are crucial for optimizing patient outcomes and enhancing the quality of life for those relying on these life-sustaining devices.

### 5.1. Gastrointestinal Bleeding

Gastrointestinal (GI) bleeding stands out as a frequent complication, affecting about one-third of dVAD patients [38,39,40]. The continuous flow nature of modern dVADs, altering physiological pulsatility, has been implicated in the development of arteriovenous malformations, a key contributor to GI bleeding [41,42]. Studies reveal a complex interplay between heart failure, dVAD support, and altered angiogenesis, leading to an increased risk of bleeding [43,44,45]. Management strategies remain in early stages, with limited evidence-based treatments being currently available.

### 5.2. Stroke

Stroke, both ischemic and hemorrhagic, represents a serious risk associated with dVADs, leading to substantial morbidity and mortality [46]. The risk of stroke has been observed to be bimodal, with peaks immediately post-implantation and again several months later. Various studies have highlighted the differences in stroke rates among different dVAD types, underlining the importance of device selection and individualized patient management [47,48,49]. Understanding and mitigating stroke risk factors are essential components of comprehensive dVAD care.

### 5.3. Device Thrombosis and Driveline Infections

Device thrombosis has emerged as a significant concern, particularly with specific dVAD models. System replacement is considered as the final therapeutic option for severe thrombosis and subsequent malfunction. It usually considered as the primary endpoint in most clinical studies of dVADs. A large-scale study of additional dVAD replacement for thrombosis and malfunction is required to evaluate the outcomes of intervention. The abrupt increase in early pump thrombosis rates observed in certain devices underscores the need for diligent monitoring and timely intervention. Recent advancements, like the development of fully magnetically levitated pumps, have shown promise in reducing the incidence of thrombosis, marking a significant step forward in dVAD technology [47,49].

Despite technological advancements, driveline infections continue to be a significant concern, affecting patient quality of life and overall device success [50,51,52]. Attempts have been made to minimize this problem, where LionHeart LVD-2000 by Arrow International and Pennsylvania University have gained remarkable progress. It applied a completely implanted left ventricular support system and controlled driveline infections in principle [53]. Although it has been removed from the market unfortunately, the feasibility of entire implantation has been confirmed. The development of transcutaneous energy transfer systems could revolutionize dVADs by eliminating the need for drivelines, potentially reducing the risk of infection.

### 5.4. Right Ventricular Failure

Right ventricular failure post-dVAD implantation remains a complex and challenging complication [51,54]. Its incidence varies across different device types and patient populations, and its management requires a multidisciplinary approach. The advancement of durable biventricular support systems may provide new avenues for addressing this complication [50].

## 6. Clinical Outcomes of Durable Ventricular Assist Device Recipients

With the revolution of dVAD technology, survival probabilities after implantation increased in 2015–2019 compared to the previous era, against the background of a worse preoperative status of recipients. Although 1-year survival rates reached 82.3% and the median survival surpassed 4.5 years, adverse events still had a remarkable occurrence rate. A total of 72% of patients were rehospitalized and 41% suffered from infection. Disparities among patients before surgery, including age, obesity, race, cardiovascular background, caregiving, and even the pandemic of COVID-19, have been considered to contribute to the different outcomes after surgery.

### 6.1. Age

One study by Emerson et al., featured in the *Journal of the American College of Cardiology*, meticulously explored the outcomes of elderly patients undergoing durable left ventricular assist device therapy. This comprehensive analysis spanned from January 2010 to March 2020, encompassing a large cohort of 24,408 patients with a median age of 57 years [55].

The study yielded insightful findings, particularly concerning the survival rates across different age groups. It was noted that the 5-year mortality rates for patients under 65, in the range of 65–75, and over 75 years were 33.5%, 54.4%, and 66.2%, respectively. Interestingly, transplantation rates were markedly higher in younger patients compared to the older cohorts. A pivotal observation was the improved survival for patients over 75 years, with a notable increment in 3-year survival rates across different periods: 43% in 2010–2012, 46.3% in 2013–2016, and 55.7% in 2017–2020 [55].

Despite a reduced overall survival rate when compared to younger patients, elderly patients receiving dVADs demonstrated similar enhancements in quality of life and functional capacity, coupled with a lower rate of major late complications. The study highlighted the substantial underutilization of durable LVAD therapy in the elderly, despite the demonstrated benefits and the increasing prevalence of advanced heart failure with advancing age.

### 6.2. Obesity

Obesity is usually recognized as a risk factor for diverse cardiovascular diseases; however, its effect in dVAD therapy seems to be more complex. In a study examining the outcomes of durable left ventricular assist device (LVAD) implantation in patients with extreme obesity, 252 patients were analyzed, with 30 (11.9%) meeting the criteria for extreme obesity [56]. Interestingly, patients with extreme obesity were found to be significantly younger (median age of 47 years) compared to their less obese counterparts (median age of 60 years) and had fewer prior sternotomies.

A higher incidence of pump thrombosis in extremely obese patients (30%) was observed compared to those with a lower body mass index (BMI) (9.0%). Additionally, the rate of stage 2/3 acute kidney injury was also higher in the extremely obese group (46.7%) compared to the less obese group (27.0%). However, despite these increased risks, there were no significant differences in 30-day or 1-year survival rates between extremely obese patients and those who were not extremely obese, even after adjusting for age and clinical factors [56].

The conclusion drawn from this study was that extreme obesity does not seem to increase the risk of mortality in patients undergoing dVAD implantation. However, the association with a higher risk of pump thrombosis indicates that extremely obese patients might require more attentive care to mitigate the need for urgent device exchange.

### 6.3. Race

A comprehensive study focused on the impact of race on the utilization of dVADs examined the outcomes for 702 patients, comprising 60.9% White and 34.1% Black individuals [57].

No statistically significant difference was found in the rates of dVAD implantation between Black and White patients after conducting a multivariate analysis that adjusted for variables such as age, sex, socioeconomic status, and clinical factors. Specifically, the odds ratio (OR) for implantation in Black patients compared to White patients was 0.68, with a 95% confidence interval of 0.45–1.04 and a *p*-value of 0.074.

This result suggests that racial disparities in the rate of LVAD implantation were not evident when controlling for other significant factors. However, the necessity for larger, prospective studies to further validate these findings was highlighted and the comprehensive understanding of the impact of race on the utilization of dVADs should be validated.

### 6.4. Congenital Heart Disease

The outcomes of patients with congenital heart disease (CHD) who underwent dVAD implantation present unique challenges and varied results [58,59,60]. The Pediatric Interagency Registry for Mechanical Circulatory Support (PEDIMACS) reported that, of 471 pediatric patients with VADs implanted from 2012 to 2017, 108 (24%) had CHD. This included 45 biventricular and 63 single-ventricle VAD-supported children [61].

A significant finding from these studies is the increased mortality and decreased transplantation rates in children with CHD on VADs. Compared to those with anatomically normal hearts, children with CHD on VADs have over a threefold increased mortality and only half the rate of transplantation [59].

Berlin Heart EXCOR, the first pediatric-specific VAD approved by the FDA, has been extensively used in patients with CHD [62,63,64]. This usage is likely due to the device’s broad range of sizes, accommodating even very small children. However, the overall mortality among pediatric patients with CHD on EXCOR support is high at 47%, with particularly high mortality (93%) in infants with CHD weighing less than 5 kg [65].

In summary, while VAD therapy in CHD patients presents challenges, including higher mortality and lower transplantation rates, especially in smaller, younger patients, certain VAD types demonstrate more favorable outcomes. These findings underscore the need for the careful selection and management of VAD therapy in the CHD population.

### 6.5. Caregiving after Surgery

In the context of dVAD implantation, the stability and quality of caregiver relationships pose an impact patient outcomes. Patients with constant caregivers, particularly spouses living in the same household, tend to experience better post-implant outcomes [66,67]. This reflects the crucial role of close and convenient caregiver–patient relationships in the effective management of dVADs.

When caregivers, especially spouses, maintain their roles throughout the dVAD support period, patients generally show improved recovery and management post-implantation. Conversely, patients who face a change in caregiver status, such as the withdrawal of a caregiver, are more likely to encounter challenges, including higher rates of 30-day post-implant readmissions [67]. This highlights the importance of consistent and reliable caregiving in the post-implant recovery phase.

An interesting aspect is the feasibility of self-care for certain dVAD patients. A specific subset of patients, often younger and more independent before implantation, can achieve outcomes comparable to those with stable caregivers, even without a designated caregiver [66]. This indicates that self-management can be an effective approach for select patients, underscoring the need for the personalized evaluation of patient capabilities and support systems in dVAD implantation.

Overall, the presence of a stable, supportive caregiving environment, particularly involving cohabitating spouses, is associated with better outcomes following dVAD implantation. However, the potential for self-care in certain patient groups suggests the importance of individualized strategies in both patient and caregiver selection to optimize outcomes in dVAD support.

### 6.6. COVID-19 Pandemic

The COVID-19 pandemic has notably impacted patients on long-term MCS, such as those with dVADs. Due to the presence of hardware, driveline exit sites, and functionally impaired immunity, this patient group inherently faces a higher risk of infections.

According to the Trans-CoV-VAD registry, a multicenter registry of LVAD and cardiac transplant patients with confirmed COVID-19 in the United States, no significant differences were observed in the racial or gender breakdown of COVID-19 cases compared to the total population receiving a ventricular assist device. However, the outcomes for these patients were concerning [68]. Of the 40 COVID-19-positive patients in the registry, 60% required hospitalization, and 25% developed critical illness. Alarmingly, the case fatality rate was high at 20%, with death occurring at a median of 22 days after admission.

Patients with advanced HF in need of LVAD implantation are particularly vulnerable. This vulnerability is compounded by the pandemic, as these patients may face longer recovery times due to the limited availability of rehabilitation options amidst COVID-19 precautions and healthcare resource constraints.

## 7. Development and Application of Durable Ventricular Assist Devices in China

### 7.1. Current Development of dVAD Technology in China

Chinese advancements in artificial hearts, particularly dVADs, have shown significant progress in recent years. Notable developments include the EVAHEART I (Yongren Heart), CH-VAD, HeartCon, and CorHeart 6 (Table 1).

Introduced as China’s first clinically approved artificial heart, EVAHEART I uses liquid suspension technology to produce a physiological pulsatile flow, offering better biocompatibility and reduced risks of thrombosis and bleeding compared to some international counterparts. It was first approved for clinical trials in 2017, marking a significant milestone in China’s dVAD landscape [69].

CH-VAD is a fully magnetically suspended artificial heart developed jointly by Fuwai Hospital and Tongxin Medical, representing a breakthrough in miniaturization, being smaller than contemporary devices, including HeartMate III. Its clinical trials began in 2017, and market approval was obtained in late 2020 [70,71,75].

HeartCon was developed in 2009, integrating dual-suspension magnetic-fluid technology and demonstrating advantages in size, weight, and heat generation. HeartCon overcame significant challenges in tissue compatibility and was clinically trialed in 2020 [72,73].

CorHeart 6 is noted for being the lightest and smallest implantable magnetic levitation centrifugal artificial heart globally, exhibiting excellent blood compatibility and low power consumption. Its first successful implantation was in 2021, followed by multicenter clinical trials. The outcomes are expected and may provide a promising future for lightweight dVADs [73,74].

### 7.2. Current State of Application, Challenges, and Future Directions

The high prevalence of heart failure in China, estimated at around 8.9 million patients in 2021, underscores a substantial need for dVADs [76]. A number a Chinese companies have joined the development and commercialization of dVADs internationally. Despite achievements in technology, China’s journey in the dVAD domain started relatively slow compared to developed nations. The development of China’s dVADs started in the early 1990s. Li et al., Chang et al., and Liu et al. have made substantially progress in long-term VADs, including the axial flow type and centrifugal type [77,78]. Although the results in vitro and in sheep seemed to be promising, their products were limited in animal experiments and pre-clinical studies [78]. The shifting trends of clinical implantation increased from the first decade of 21th century onwards, with the maturity of magnetic-liquid suspension blood pump technology. The primary outcomes of domestic dVADs satisfied the expectations. One study conducted by Fuwai Hospital obtained a 90% two-year survival probability in 70 end-stage heart failure patients [79]. The short-form health survey score showed that the quality of life of patients was significantly improved after operation, and there were no serious adverse events of blood compatibility, such as pump thrombosis, stroke, and gastrointestinal bleeding. Several clinical studies are also undergoing for the further exploration of domestic dVADs’ curative effects (Table 2). Compared to heart transplantation, the current data show a higher proportion of survival with 90% in two years from Fuwai Hospital versus 84.34% in one year from ISHLT. Moreover, heart transplants always incur high costs (USD 990,000/case in the U.S. and RMB 300,000/case in China) and long-term use of immunosuppressive agents (approximately RMB 5000/month in China) [80]. The better development and boarder commercial application of dVADs will benefit patients regarding both prognosis and financial costs. From 2017 to 2022, the number of hospitals that were qualified for dVAD implantation increased from 1 to 33, and the cases of implantation reached over 200 over the country. However, most of the current applications are centered around BTT and Bridge to Decision (BTD), with a notable lack of research in DT [81].

Hong Kong’s experience with dVADs started in 2010. By 2020, Hong Kong had performed LVAD implantations in 95 patients, increasingly using dVADs over heart transplants. The one-year survival probabilities reached 84% and five-year survival probabilities reached 69%, significantly higher than the international average level. However, differently from the mainland, only BTT dVADs are supported by the Hong Kong healthcare system and clinical trials have limited development in this Special Administrative Region [82].

The slower overall development, high costs, and nascent clinical application status of the domestic trend present significant challenges in China. The challenges that the commercialization of dVADs in China faces may be attributed to inadequate infrastructure and the need for extensive collaborative efforts among various stakeholders [82]. Complications, such as bleeding, thrombosis, cable infection, and stroke, are still the most important challenges for the long-term management of ventricular assist devices. The incidence of gastrointestinal bleeding and stroke in domestic patients is significantly lower than that in foreign patients, but the incidence of cable infection and arrhythmia is higher. This suggests that domestic practitioners should invest more efforts in the research and development of fully implanted wireless blood pumps, improving biomaterials, and improving blood compatibility [79]. With the great advanced HF population requiring dVADs for BTT or DT and the general process in the medical industry, both domestic devices’ research development and international collaboration face a bright future. Moreover, a recent study about dVADs in China remains at the clinical trial stage; large-scale clinical and epidemiological research after feasibility confirmation tends to be arranged on schedule.

## 8. Conclusions

The development and use of durable ventricular assist devices (VADs) are critical in the management of advanced heart failure, especially when heart transplantation is not feasible due to limited donors or unqualified patients. This review provided a comprehensive overview of various VADs, their development, and future prospects.

Since Debakey et al. implanted the first gas-energized pump in 1966, dVADs have undergone three generations as an important category of VADs. The transition from the first generation of pulsatile dVADs to the second generation of continuous flow devices has resulted in improvements in size, reliability, durability, energy efficiency, thrombogenicity, and ease of implantation. However, challenges, such as the wear and tear of the device components, the lack of pulsatile blood flow, excessive anticoagulation requirements, and driveline infections, created limitations. To address these issues, third-generation fully supported devices were developed, featuring suspended, magnetically levitated impellers for improved durability and reduced frictional heat. Technological advances and product iterations have significantly improved the probabilities of patient survival and quality of life after surgery.

With the irreversible increase in the number of people with end-stage heart failure combined with an increased donor–recipient imbalance, strategies for the use of dVADs have changed significantly. More patients are receiving dVADs for DTs, and the proportion of BTT dVADs has maintained an upward trend.

In the future, the development of dVAD technology should focus on reducing thrombosis, minimizing blood damage, decreasing implantation damage, and adapting to the changing demands of blood flow in the body. Ongoing research and clinical trials are critical in shaping the future outlook of mechanical circulatory support.

## Figures and Tables

**Table 1:** Basic parameter table of durable ventricular assist devices made in China.

Product Name	Weight	State	Rotation Speed	Maximum Flow	Structure and Composition/Main Components	Scope of Application/Intended Use	Registration Certificate Number	Name of Registrant	Relevant Research
EvaHeart I	420 g	On the market	1800–2200 r/min	20 L/min	Composed of the body parts and surgical tools. The body parts include pumping blood components (including pump line) into the blood vessels, controller, the controller-connected components, pure water-sealing components, pure water seal flow cleaning component, AC/DC adapter, battery, charger, emergency batteries, the standby controller, external monitor and external monitor base, external monitor connection cables, key operation, surgery tools including tunnel knife, punch, wrench, and dissector.	It is used to provide mechanical support for the blood circulation of patients with advanced refractory left heart failure, that is, before heart transplantation or transitional treatment to restore heart function and long-term treatment. It can be used by medical institutions with heart transplantation conditions and comprehensive nursing ability after operation.	20193120603	Chongqing Yongrenxin Medical Equipment Co., Ltd. (Chongqing, China)	[69]
CH-VAD	180 g	On the market	2500–31,000 r/min	11 L/min	The blood pump, in vitro controller, rechargeable lithium batteries, battery charger, communication isolation module, monitor, surgical tools, and shower bag.	The product is used in conjunction with specific artificial blood vessels to provide mechanical support for the blood circulation of patients with advanced refractory left heart failure before heart transplantation or as a transitional therapy to restore heart function. It can be used by medical institutions with heart transplantation conditions and comprehensive nursing ability after operation.	20213120987	Suzhou Tongxin Medical Technology Co., Ltd. (Suzhou, China)	[70,71]
HeartCon	180 g	On the market	2000~3600 r/min	10 L/min	Products by the implant components (pump (model HeartCon), ventricular suture ring, and artificial blood vessel protection frame), surgical tools (heart-opening tool, tightening tool, percutaneous lead traction tool, inlet tube protective cap, surgical percutaneous lead extension cable, and pump cable extension cable), and external components.	The product is used in conjunction with specific artificial blood vessels to provide mechanical support for the blood circulation of patients with advanced refractory left heart failure before heart transplantation or as a transitional therapy to restore heart function. It can be used by medical institutions with heart transplantation conditions and comprehensive nursing ability after operation.	20223120892	Aerospace Taixin Technology Co., Ltd. (Tianjin, China)	[72,73]
CorHeart6	90 g	On the market	2400~3000 r/min	N/A	It is composed of implanted components, external components, and surgical accessories. The implanted components include the left ventricular assist pump (Corheart 6), apical ring (the apical suture ring, CH-ACC-01), outlet tube (CH-OF-01), and the left ventricular assist pump. The external components include a connector cover, a controller, a rechargeable lithium battery pack, a power adapter, a charging dock, a communication adapter, monitor, carry-on bag, shower package, surgical accessories including hole opener, pull A knife, connectors, and gland.	It can be used in combination with specific artificial blood vessels to provide mechanical support for the blood circulation of patients with advanced refractory left heart failure before heart transplantation or as a transitional therapy to restore heart function. It can be used by medical institutions with heart transplantation conditions and comprehensive nursing ability after operation.	20233120716	Shenzhen Core Medical Technology Co., Ltd. (Shenzhen, China)	[74]

**Table 2:** Clinical studies of durable ventricular assist devices in China registered on *clinicaltrials.gov*.

Rank	NCT Number	Title	Status	Conditions	Interventions	Enrollment	Study Type	Locations	URL
1	NCT05928273	Corheart 6 LVAS LTFU	Completed	End-stage heart failure	Device: Corheart 6 Left Ventricular Assist System	50	Interventional	Fuwai Hospital, Chinese Academy of Medical Sciences, Beijing, China	https://ClinicalTrials.gov/show/NCT05928273 (accessed on 16 January 2024).
2	NCT05353816	Corheart 6 Left Ventricular Assist System Prospective, Multicenter, Single-arm Clinical Evaluation Trial	Completed	Critical or chronic advanced heart failure	Device: Intervention/treatment	50	Interventional	Shenzhen Core Medical Technology Co.,Ltd., Shenzhen, Guangdong, China	https://ClinicalTrials.gov/show/NCT05353816 (accessed on 16 January 2024).
3	NCT04464356	Application of Emergent Peripheral Mechanical Circulatory Support in Transcatheter Aortic Valve Replacement for 6 Patients	Completed	Transcatheter aortic valve replacement|mechanical circulatory support	Device: mechanical circulatory support	6	Observational	Second Affiliated Hospital, School of Medicine, Zhejiang University, Hangzhou, Zhejiang, China	https://ClinicalTrials.gov/show/NCT04464356 (accessed on 16 January 2024).
4	NCT02731794	On-pump Beating Coronary Artery Bypass Grafting by Ventricular Assist	Unknown status	Coronary artery bypass	Device: left ventricular assist|Procedure: Biventricular assist	70	Interventional	Henan Provincial People’ Hospital, Zhengzhou, Henan, China	https://ClinicalTrials.gov/show/NCT02731794 (accessed on 16 January 2024).
5	NCT06127927	Evaluation the Efficacy and Safety of an Interventional Left Ventricular Assist System for Hemodynamic Support in Patients With Cardiogenic Shock	Recruiting	Cardiogenic shock	Device: Impella	66	Interventional	Department of Cardiovascular Surgery, Fuzhou, Fujian, China|Fujian Medical University Union Hospital, Fuzhou, Fujian, China	https://ClinicalTrials.gov/show/NCT06127927 (accessed on 16 January 2024).

## References

[1] Tsao C.W., Aday A.W., Almarzooq Z.I., Anderson C., Arora P., Avery C.L., Baker-Smith C.M., Beaton A.Z., Boehme A.K., Buxton A.E. (2023). Heart disease and stroke statistics-2023 update: A report from the american heart association. Circulation.

[2] Yancy C.W., Jessup M., Bozkurt B., Butler J., Casey D.J., Drazner M.H., Fonarow G.C., Geraci S.A., Horwich T., Januzzi J.L. (2013). 2013 ACCF/AHA guideline for the management of heart failure: A report of the American College of Cardiology Foundation/American Heart Association Task Force on Practice Guidelines. J. Am. Coll. Cardiol..

[3] Ambrosy A.P., Fonarow G.C., Butler J., Chioncel O., Greene S.J., Vaduganathan M., Nodari S., Lam C., Sato N., Shah A.N. (2014). The global health and economic burden of hospitalizations for heart failure: Lessons learned from hospitalized heart failure registries. J. Am. Coll. Cardiol..

[4] Ma L.Y., Chen W.W., Gao R.L., Liu L.S., Zhu M.L., Wang Y.J., Wu Z.S., Li H.J., Gu D.F., Yang Y.J. (2020). China cardiovascular diseases report 2018: An updated summary. J. Geriatr. Cardiol..

[5] Mclarty A. (2015). Mechanical circulatory support and the role of LVADs in heart failure therapy. Clin. Med. Insights. Cardiol..

[6] Kirklin J.K., Naftel D.C., Pagani F.D., Kormos R.L., Stevenson L., Miller M., Young J.B. (2012). Long-term mechanical circulatory support (destination therapy): On track to compete with heart transplantation?. J. Thorac. Cardiovasc. Surg..

[7] Wilmot I., Lorts A., Morales D. (2013). Pediatric mechanical circulatory support. Korean J. Thorac. Cardiovasc. Surg..

[8] Dichiacchio L., Heiler J., Kagawa H., Selzman C.H., Goodwin M.L. (2024). Minimally Invasive Direct Aortic Implantation of Impella 5.5 as Bridge to Transplant: A Case Report. Transplantation Proceedings.

[9] Wampler R., Frazier O.H. (2019). The hemopump, the first intravascular ventricular assist device. ASAIO J..

[10] King K., Grazette L.P., Paltoo D.N., Mcdevitt J.T., Sia S.K., Barrett P.M., Apple F.S., Gurbel P.A., Weissleder R., Leeds H. (2016). Point-of-Care technologies for precision cardiovascular care and clinical research: National heart, lung, and blood institute working group. JACC Basic Transl. Sci..

[11] Perry A.S., Li S. (2021). Optimal threshold of left ventricular ejection fraction for aortic valve replacement in asymptomatic severe aortic stenosis: A systematic review and Meta-Analysis. J. Am. Heart Assoc..

[12] Kilic A., Katz J.N., Joseph S.M., Brisco-Bacik M.A., Uriel N., Lima B., Agarwal R., Bharmi R., Farrar D.J., Lee S. (2019). Changes in pulmonary artery pressure before and after left ventricular assist device implantation in patients utilizing remote haemodynamic monitoring. ESC Heart Fail.

[13] Hastings F.W. (1968). Contracting policies of artificial heart program. Science.

[14] Prinzing A., Herold U., Berkefeld A., Krane M., Lange R., Voss B. (2016). Left ventricular assist devices-current state and perspectives. J. Thorac. Dis..

[15] Debakey M.E. (1971). Left ventricular bypass pump for cardiac assistance. Clinical experience. Am. J. Cardiol..

[16] Etz C., Welp H., Tjan T.D., Krasemann T., Schmidt C., Scheld H.H., Schmid C. (2004). Successful long-term bridge to transplant in a 5-year-old boy with the EXCOR left ventricular assist device. Thorac. Cardiovasc. Surg..

[17] Myers T.J., Mcgee M.G., Zeluff B.J., Radovancevic B., Frazier O.H. (1991). Frequency and significance of infections in patients receiving prolonged LVAD support. ASAIO Trans..

[18] Birks E.J., Tansley P.D., Yacoub M.H., Bowles C.T., Hipkin M., Hardy J., Banner N.R., Khaghani A. (2004). Incidence and clinical management of life-threatening left ventricular assist device failure. J. Heart Lung. Transpl..

[19] Han J., Trumble D.R. (2019). Cardiac assist devices: Early concepts, current technologies, and future innovations. Bioengineering.

[20] Griffith B.P., Kormos R.L., Borovetz H.S., Litwak K., Antaki J.F., Poirier V.L., Butler K.C. (2001). HeartMate II left ventricular assist system: From concept to first clinical use. Ann. Thorac. Surg..

[21] Macris M.P., Myers T.J., Jarvik R., Robinson J.L., Fuqua J.M., Parnis S.M., Frazier O.H. (1994). In vivo evaluation of an intraventricular electric axial flow pump for left ventricular assistance. ASAIO J..

[22] Moazami N., Fukamachi K., Kobayashi M., Smedira N.G., Hoercher K.J., Massiello A., Lee S., Horvath D.J., Starling R.C. (2013). Axial and centrifugal continuous-flow rotary pumps: A translation from pump mechanics to clinical practice. J. Heart Lung Transpl..

[23] Krabatsch T., Schweiger M., Dandel M., Stepanenko A., Drews T., Potapov E., Pasic M., Weng Y.G., Huebler M., Hetzer R. (2011). Is bridge to recovery more likely with pulsatile left ventricular assist devices than with nonpulsatile-flow systems?. Ann. Thorac. Surg..

[24] Molina E.J., Shah P., Kiernan M.S., Cornwell W.R., Copeland H., Takeda K., Fernandez F.G., Badhwar V., Habib R.H., Jacobs J.P. (2021). The Society of Thoracic Surgeons Intermacs 2020 Annual Report. Ann. Thorac. Surg..

[25] Schmitto J.D., Hanke J.S., Rojas S.V., Avsar M., Haverich A. (2015). First implantation in man of a new magnetically levitated left ventricular assist device (HeartMate III). J. Heart Lung Transpl..

[26] Mehra M.R., Goldstein D.J., Cleveland J.C., Cowger J.A., Hall S., Salerno C.T., Naka Y., Horstmanshof D., Chuang J., Wang A. (2022). Five-Year Outcomes in Patients with Fully Magnetically Levitated vs Axial-Flow Left Ventricular Assist Devices in the MOMENTUM 3 Randomized Trial. JAMA.

[27] Schramm R., Zittermann A., Morshuis M., Schoenbrodt M., von Roessing E., von Dossow V., Koster A., Fox H., Hakim-Meibodi K., Gummert J.F. (2020). Comparing short-term outcome after implantation of the HeartWare(R) HVAD(R) and the Abbott(R) HeartMate 3(R). ESC Heart Fail.

[28] Vriz O., Mushtaq A., Shaik A., El-Shaer A., Feras K., Eltayeb A., Alsergnai H., Kholaif N., Al H.M., Albert-Brotons D. (2022). Reciprocal interferences of the left ventricular assist device and the aortic valve competence. Front. Cardiovasc. Med..

[29] Letsou G.V., Pate T.D., Gohean J.R., Kurusz M., Longoria R.G., Kaiser L., Smalling R.W. (2010). Improved left ventricular unloading and circulatory support with synchronized pulsatile left ventricular assistance compared with continuous-flow left ventricular assistance in an acute porcine left ventricular failure model. J. Thorac. Cardiovasc. Surg..

[30] Mcgee E.J., Danter M., Strueber M., Mahr C., Mokadam N.A., Wieselthaler G., Klein L., Lee S., Boeve T., Maltais S. (2019). Evaluation of a lateral thoracotomy implant approach for a centrifugal-flow left ventricular assist device: The LATERAL clinical trial. J. Heart Lung Transpl..

[31] Cho S.M., Mehaffey J.H., Meyers S.L., Cantor R.S., Starling R.C., Kirklin J.K., Jacobs J.P., Kern J., Uchino K., Yarboro L.T. (2021). Cerebrovascular events in patients with Centrifugal-Flow left ventricular assist devices: Propensity Score-Matched analysis from the intermacs registry. Circulation.

[32] Jorde U.P., Saeed O., Koehl D., Morris A.A., Wood K.L., Meyer D.M., Cantor R., Jacobs J.P., Kirklin J.K., Pagani F.D. (2024). The Society of Thoracic Surgeons Intermacs 2023 Annual Report: Focus on Magnetically Levitated Devices. Ann. Thorac. Surg..

[33] Varshney A.S., Defilippis E.M., Cowger J.A., Netuka I., Pinney S.P., Givertz M.M. (2022). Trends and outcomes of left ventricular assist device therapy: JACC focus seminar. J. Am. Coll. Cardiol..

[34] Stehlik J., Edwards L.B., Kucheryavaya A.Y., Benden C., Christie J.D., Dipchand A.I., Dobbels F., Kirk R., Rahmel A.O., Hertz M.I. (2012). The Registry of the International Society for Heart and Lung Transplantation: 29th official adult heart transplant report-2012. J. Heart Lung Transpl..

[35] Kirklin J.K., Pagani F.D., Kormos R.L., Stevenson L.W., Blume E.D., Myers S.L., Miller M.A., Baldwin J.T., Young J.B., Naftel D.C. (2017). Eighth annual INTERMACS report: Special focus on framing the impact of adverse events. J. Heart Lung Transpl..

[36] Varshney A.S., Hirji S.A., Givertz M.M. (2020). Outcomes in the 2018 UNOS donor heart allocation system: A perspective on disparate analyses. J. Heart Lung Transpl..

[37] Goldstein D.J., Naka Y., Horstmanshof D., Ravichandran A.K., Schroder J., Ransom J., Itoh A., Uriel N., Cleveland J.J., Raval N.Y. (2020). Association of clinical outcomes with left ventricular assist device use by bridge to transplant or destination therapy intent: The multicenter study of MagLev technology in patients undergoing mechanical circulatory support therapy with HeartMate 3 (MOMENTUM 3) randomized clinical trial. JAMA Cardiol..

[38] Makay B., Turkyilmaz Z., Duman M., Unsal E. (2009). Mean platelet volume in Henoch-Schonlein purpura: Relationship to gastrointestinal bleeding. Clin. Rheumatol..

[39] Wang S., Griffith B.P., Wu Z.J. (2021). Device-Induced hemostatic disorders in mechanically assisted circulation. Clin. Appl. Thromb. Hemost..

[40] Ahsan I., Faraz A., Mehmood A., Ullah W., Ghani A.R. (2019). Clinical approach to manage gastrointestinal bleeding with a left ventricular assist device (LVAD). Cureus.

[41] Gurvits G.E., Fradkov E. (2017). Bleeding with the artificial heart: Gastrointestinal hemorrhage in CF-LVAD patients. World J. Gastroenterol..

[42] Demirozu Z.T., Radovancevic R., Hochman L.F., Gregoric I.D., Letsou G.V., Kar B., Bogaev R.C., Frazier O.H. (2011). Arteriovenous malformation and gastrointestinal bleeding in patients with the HeartMate II left ventricular assist device. J. Heart Lung Transpl..

[43] Patel S.R., Madan S., Saeed O., Algodi M., Luke A., Gibber M., Goldstein D.J., Jorde U.P. (2016). Association of nasal mucosal vascular alterations, gastrointestinal arteriovenous malformations, and bleeding in patients with Continuous-Flow left ventricular assist devices. JACC Heart Fail..

[44] Tabit C.E., Chen P., Kim G.H., Fedson S.E., Sayer G., Coplan M.J., Jeevanandam V., Uriel N., Liao J.K. (2016). Elevated angiopoietin-2 level in patients with Continuous-Flow left ventricular assist devices leads to altered angiogenesis and is associated with higher nonsurgical bleeding. Circulation.

[45] Tabit C.E., Coplan M.J., Chen P., Jeevanandam V., Uriel N., Liao J.K. (2018). Tumor necrosis factor-alpha levels and non-surgical bleeding in continuous-flow left ventricular assist devices. J. Heart Lung Transpl..

[46] Frontera J.A., Starling R., Cho S.M., Nowacki A.S., Uchino K., Hussain M.S., Mountis M., Moazami N. (2017). Risk factors, mortality, and timing of ischemic and hemorrhagic stroke with left ventricular assist devices. J. Heart Lung Transpl..

[47] Katz J.N., Adamson R.M., John R., Tatooles A., Sundareswaran K., Kallel F., Farrar D.J., Jorde U.P. (2015). Safety of reduced anti-thrombotic strategies in HeartMate II patients: A one-year analysis of the US-TRACE Study. J. Heart Lung Transpl..

[48] Teuteberg J.J., Slaughter M.S., Rogers J.G., Mcgee E.C., Pagani F.D., Gordon R., Rame E., Acker M., Kormos R.L., Salerno C. (2015). The HVAD left ventricular assist device: Risk factors for neurological events and risk mitigation strategies. JACC Heart Fail..

[49] Maltais S., Kilic A., Nathan S., Keebler M., Emani S., Ransom J., Katz J.N., Sheridan B., Brieke A., Egnaczyk G. (2017). PREVENtion of HeartMate II Pump Thrombosis through Clinical Management: The PREVENT multi-center study. J. Heart Lung Transpl..

[50] Rogers J.G., Pagani F.D., Tatooles A.J., Bhat G., Slaughter M.S., Birks E.J., Boyce S.W., Najjar S.S., Jeevanandam V., Anderson A.S. (2017). Intrapericardial left ventricular assist device for advanced heart failure. N. Engl. J. Med..

[51] Mehra M.R., Naka Y., Uriel N., Goldstein D.J., Cleveland J.J., Colombo P.C., Walsh M.N., Milano C.A., Patel C.B., Jorde U.P. (2017). A fully magnetically levitated circulatory pump for advanced heart failure. N. Engl. J. Med..

[52] Grinstein J., Rodgers D., Kalantari S., Sayer G., Kim G.H., Sarswat N., Adatya S., Ota T., Jeevanandam V., Burkhoff D. (2018). HVAD waveform analysis as a noninvasive marker of pulmonary capillary wedge pressure: A first step toward the development of a smart left ventricular assist device pump. ASAIO J..

[53] Mehta S.M., Pae W.J., Rosenberg G., Snyder A.J., Weiss W.J., Lewis J.P., Frank D.J., Thompson J.J., Pierce W.S. (2001). The LionHeart LVD-2000: A completely implanted left ventricular assist device for chronic circulatory support. Ann. Thorac. Surg..

[54] Lampert B.C., Teuteberg J.J. (2015). Right ventricular failure after left ventricular assist devices. J. Heart Lung Transpl..

[55] Emerson D., Chikwe J., Catarino P., Hassanein M., Deng L., Cantor R.S., Roach A., Cole R., Esmailian F., Kobashigawa J. (2021). Contemporary left ventricular assist device outcomes in an aging population: An STS INTERMACS analysis. J. Am. Coll. Cardiol..

[56] Lee A.Y., Tecson K.M., Lima B., Shaikh A.F., Collier J., Still S., Baxter R., Dimaio J.M., Felius J., Carey S.A. (2019). Durable left ventricular assist device implantation in extremely obese heart failure patients. Artif. Organs..

[57] Jones M.M., Mcelroy L.M., Mirreh M., Fuller M., Schroeder R., Ghadimi K., Devore A., Patel C.B., Black-Maier E., Bartz R. (2022). The impact of race on utilization of durable left ventricular assist device therapy in patients with advanced heart failure. J. Card. Surg..

[58] Villa C.R., Khan M.S., Zafar F., Morales D., Lorts A. (2017). United states trends in pediatric ventricular assist implantation as bridge to transplantation. ASAIO J..

[59] Peng D.M., Koehl D.A., Cantor R.S., Mcmillan K.N., Barnes A.P., Mcconnell P.I., Jordan J., Andersen N.D., St L.J., Maeda K. (2019). Outcomes of children with congenital heart disease implanted with ventricular assist devices: An analysis of the Pediatric Interagency Registry for Mechanical Circulatory Support (Pedimacs). J. Heart Lung Transpl..

[60] Rossano J.W., Woods R.K., Berger S., Gaynor J.W., Ghanayem N., Morales D.L., Ravishankar C., Mitchell M.E., Shah T.K., Mahr C. (2013). Mechanical support as failure intervention in patients with cavopulmonary shunts (MFICS): Rationale and aims of a new registry of mechanical circulatory support in single ventricle patients. Congenit. Heart Dis..

[61] Dipchand A.I., Kirk R., Naftel D.C., Pruitt E., Blume E.D., Morrow R., Rosenthal D., Auerbach S., Richmond M.E., Kirklin J.K. (2018). Ventricular assist device support as a bridge to transplantation in pediatric patients. J. Am. Coll. Cardiol..

[62] Jurmann M.J., Weng Y., Drews T., Pasic M., Hennig E., Hetzer R. (2004). Permanent mechanical circulatory support in patients of advanced age. Eur. J. Cardiothorac. Surg..

[63] Conway J., Amdani S., Morales D., Lorts A., Rosenthal D.N., Jacobs J.P., Rossano J., Koehl D., Kirklin J.M., Auerbach S.R. (2023). Widening care gap in VAD therapy. J. Heart Lung Transpl..

[64] Stephens N.A., Chartan C.A., Gazzaneo M.C., Thomas J.A., Das S., Mallory G.J., Melicoff E., Vogel A.M., Parker A., Hermes E. (2023). Use of Berlin EXCOR cannulas in both venovenous and venoarterial central extracorporeal membrane oxygenation configurations overcomes the problem of cannula instability while bridging infants and young children to lung transplant. JTCVS Tech..

[65] Almond C.S., Morales D.L., Blackstone E.H., Turrentine M.W., Imamura M., Massicotte M.P., Jordan L.C., Devaney E.J., Ravishankar C., Kanter K.R. (2013). Berlin Heart EXCOR pediatric ventricular assist device for bridge to heart transplantation in US children. Circulation.

[66] Koeckert M., Vining P., Reyentovich A., Katz S.D., Deanda A.J., Philipson S., Balsam L.B. (2017). Caregiver status and outcomes after durable left ventricular assist device implantation. Heart Lung.

[67] Wright G.A., Rauf A., Stoker S., Alharethi R., Kfoury A.G. (2015). Marital status and survival in left ventricular assist device patient populations. J. Heart Lung Transpl..

[68] Birati E.Y., Najjar S.S., Tedford R.J., Houston B.A., Shore S., Vorovich E., Atluri P., Urgo K., Molina M., Chambers S. (2021). Characteristics and outcomes of COVID-19 in patients on left ventricular assist device support. Circ. Heart Fail..

[69] Chen H.B., Wang X.Q., Du J., Shi J., Ji B.Y., Shi L., Shi Y.S., Zhou X.T., Yang X.H., Hu S.S. (2023). Long-term outcome of EVAHEART I implantable ventricular assist device for the treatment of end stage heart failure: Clinical 3-year follow-up results of 15 cases. Zhonghua Xin Xue Guan Bing Za Zhi.

[70] Lin C., Wu G., Liu X., Xu C., Hou X., Li H., Chen C., Yang P., Wang J., Liu Y. (2015). In vivo evaluation of an implantable magnetic suspending left ventricular assist device. Int. J. Artif. Organs..

[71] Xu C., Wu G., Liu X., He Y., Hou X., Li H., Chen C., Yang P., Lin C. (2018). Preclinical study of anticoagulation regimens in sheep after implantation of CH-VAD blood pump. Artif. Organs..

[72] Wang W., Song Y., Zhang Y., Wang Z., Liu Z., Li S., Tang Y., Liu X. (2022). Short-term effect of HeartCon left ventricular assist device on the treatment of 20 adult patients with end-stage heart failure. Zhonghua Wei Zhong Bing Ji Jiu Yi Xue.

[73] Chen L.W., Wu Q.S., Dai X.F., Dong Y., Li Q.Z., Fang G.H., Zhang G.C. (2023). Early results of left ventricular assist device implantation for the treatment of heart failure. Zhonghua Yi Xue Za Zhi.

[74] Fang P., Yang Y., Wei X., Yu S. (2023). Preclinical evaluation of the fluid dynamics and hemocompatibility of the Corheart 6 left ventricular assist device. Artif. Organs..

[75] Wang Y., Smith P.A., Handy K.M., Conger J.L., Spangler T., Lin F., Chen C., Costas G., Elgalad A., Sampaio L.C. (2020). In vivo hemodynamic evaluation of an implantable left ventricular assist device in a long-term anti-coagulation regimen. Proceedings of the 2020 42nd Annual International Conference of the IEEE Engineering in Medicine & Biology Society (EMBC).

[76] In C.T., Hu S.S. (2023). Report on cardiovascular health and diseases in China 2021: An updated summary. J. Geriatr. Cardiol..

[77] Chang Y., Gao B. (2010). Modeling and identification of an intra-aorta pump. ASAIO J..

[78] Gu K., Chang Y., Gao B., Wan F., Loisance D., Zeng Y. (2014). Development of ventricular assist devices in China: Present status, opportunities and challenges. Eur. J. Cardiothorac. Surg..

[79] Hu S.S. (2023). Surgical treatment of heart failure in China: Towards the era of artificial heart. Zhonghua Wai Ke Za Zhi.

[80] Sun Y.F., Wang Z.W., Zhang J., Cai J., Shi F., Dong N.G. (2021). Current Status of and Opinions on Heart Transplantation in China. Curr. Med. Sci..

[81] Berk Z., Zhang J., Chen Z., Tran D., Griffith B.P., Wu Z.J. (2019). Evaluation of in vitro hemolysis and platelet activation of a newly developed maglev LVAD and two clinically used LVADs with human blood. Artif. Organs..

[82] Sivathasan C., Hayward C., Jansz P., Sibal A.K., Cally H.K.L., Balakrishnan K.R., Cho Y.H., Nordin M.N., Barril J.B., Khaliel F. (2020). Durable mechanical circulatory support across the Asia-Pacific region. J. Heart Lung Transpl..

